# Frog hatchlings use early environmental cues to produce an anticipatory resource-use phenotype

**DOI:** 10.1098/rsbl.2022.0613

**Published:** 2023-03-29

**Authors:** Emily A. Harmon, Boyce Evans, David W. Pfennig

**Affiliations:** Department of Biology, University of North Carolina, Chapel Hill, NC 27599, USA

**Keywords:** developmental plasticity, anticipatory plasticity, *Spea multiplicata*

## Abstract

Developmental plasticity can occur at any life stage, but plasticity that acts early in development may give individuals a competitive edge later in life. Here, we asked if early (pre-feeding) exposure to a nutrient-rich resource impacts hatchling morphology in Mexican spadefoot toad tadpoles, *Spea multiplicata*. A distinctive carnivore morph can be induced when tadpoles eat live fairy shrimp. We investigated whether cues from shrimp––detected before individuals are capable of feeding––alter hatchling morphology such that individuals could potentially take advantage of this nutritious resource once they begin feeding. We found that hatchlings with early developmental exposure to shrimp were larger and had larger jaw muscles––traits that, at later stages, increase a tadpole's competitive ability for shrimp. These results suggest that early developmental stages can assess and respond to environmental cues by producing resource-use phenotypes appropriate for future conditions. Such anticipatory plasticity may be an important but understudied form of developmental plasticity.

## 1. Introduction

Developmental plasticity––the ability of organisms to change their phenotype in response to variation in their environment [1,2]––varies across life stages [3]. Indeed, many organisms have sensitive periods when they are receptive to environmental cues. Moreover, cues received earlier in development often affect phenotypes more than those received later [4].

Early life stages have been shown to assess and respond to their environments adaptively. For instance, some amphibians can delay or accelerate hatching based on the presence of predators or other egg threats [5–10]. These embryos exhibit ‘responsive plasticity,’ where an individual alters development in response to an environment already present. By contrast, ‘anticipatory plasticity’ occurs when environmental cues induce phenotypic changes in the expectation of an environment experienced later in life [11]. For example, in certain butterflies, environmental cues experienced by larvae or pupae generate distinctly different––but adaptive––wing phenotypes in the adults during spring versus summer [12]. Similarly, the common frog, *Rana temporaria*, responds to embryonic exposure to predator cues by hatching with defensive morphology of shorter bodies and deeper tail fins [13]. Here, we document a novel anticipatory plastic response involving resource-use plasticity.

Resource-use plasticity, where organisms produce different traits depending on their diet, occurs in diverse taxa [14–21]. Such plasticity allows organisms to alter their phenotypes to use resources more effectively while minimizing intraspecific competition [22,23]. Although resource-use plasticity often derives from responsive plasticity, responsive plasticity is limited because there is a delay between receiving the cue via diet and responding to the cue. Thus, in highly competitive environments (where resource-use plasticity often evolves), individuals that assess the environment early in development––even before foraging––could gain a competitive edge later in life. In such cases, anticipatory plasticity could lead to the expression of phenotypes that are especially well suited for acquiring *subsequent* resources.

**Figure 1.**
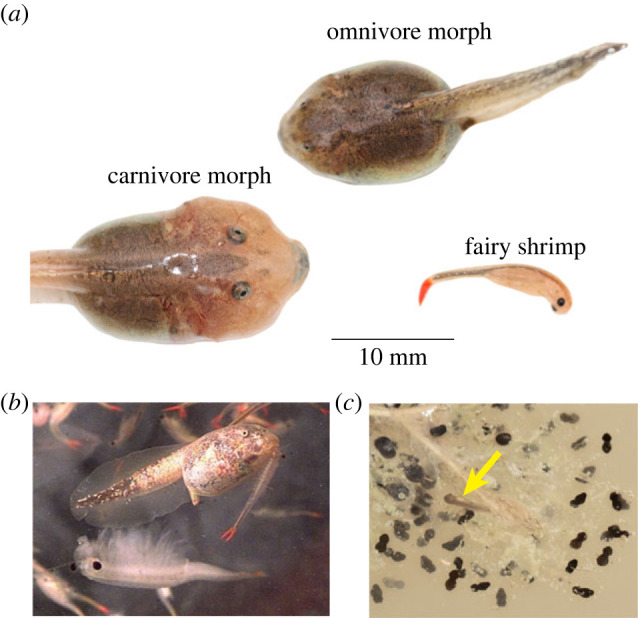
Developmental plasticity in spadefoot toad tadpoles. (*a*) *Spea multiplicata* develop into an omnivore or carnivore morph, which has features (e.g. large jaw muscles) for eating animal prey, such as fairy shrimp. (*b*) Previous studies have shown that carnivores are produced when young omnivores eat shrimp. (*c*) We investigated whether early (pre-feeding) exposure to shrimp caused hatchlings to produce more carnivore-like features (note the hatchling (yellow arrow) surrounded by embryos; hatchlings remain immobile and do not feed for 24 h after hatching).

Intense competitive interactions and resource-use plasticity are found in Mexican spadefoot toads, *Spea multiplicata*. Their tadpoles develop plastically as either an ‘omnivore’ morph, which feeds on detritus, algae and small plankton, or a ‘carnivore’ morph, which specializes on and is induced by eating animal prey, such as fairy shrimp and tadpoles (figure 1) [18,24,25]. The carnivore is characterized by larger body size, larger jaw muscles, shorter gut and serrated mouthparts, all of which facilitate the capture of large, active prey [18,24,25].

By specializing on high-nutrition animal prey, carnivores develop faster and are more likely to escape a rapidly drying pond [18,25,26]. Indeed, there is often severe competition for shrimp [25], and individuals with more extreme carnivore features (e.g. larger body size and jaw muscles) have a competitive edge [27,28]. Thus, in a rapidly drying pond, any individual that can detect shrimp and begin developing a phenotype to consume this resource would have an advantage as soon as they can feed.

We asked whether early developmental stages of *S. multiplicata* respond to resource cues. We specifically investigated whether exposing eggs to shrimp cues induced hatchlings with larger body size, larger jaw muscles, or shorter gut––traits that increase the competitive ability for shrimp [27,28] and are diagnostic of the carnivore phenotype [18,24,25]. We found that even pre-feeding developmental stages can respond to environmental cues by producing phenotypes that, at later stages, would likely enhance their ability to eat shrimp.

## 2. Material and methods

In nature, fairy shrimp hatch within 48 h of pond filling. Depending on the time of day a pond fills, *S. multiplicata* oviposit within 2–20 h of pond filling (they breed only on the night after a pond fills). Because *S. multiplicata* eggs take another 48 h to hatch, *S. multiplicata* embryos would typically be exposed to live shrimp for at least 2–20 h.

In July 2020, we mimicked natural conditions by adding fairy shrimp eggs, *Streptocephalus* sp., to 5-gallon aquaria of distilled water placed by a window. *Streptocephalus* occur in natural ponds where our frogs were collected. Their nauplii began hatching within 24 h. We started feeding the shrimp nauplii Brine Shrimp Food (Northeastern Brine Shrimp LLC) when 1 day old. We harvested shrimp from aquaria and transferred them to a tub of dechlorinated tap water. We repeated this step to rinse away residual food particles and concentrate the shrimp's density.

Approximately 20 h after the shrimp eggs were placed in water, we bred three pairs of *S. multiplicata* collected near Portal, Arizona, USA, that had been part of a laboratory colony at UNC-Chapel Hill for up to 4 years. We induced breeding by injecting adults with 0.07 ml luteinizing hormone-releasing hormone at 0.01 µg µl^−1^ and leaving the pairs in separate nursery tanks overnight. While the frogs were breeding, we filled 60 replicate plastic boxes (11 × 11 × 11 cm) with 900 ml of dechlorinated tap water. We assigned boxes to the shrimp exposure treatment or control, adding 10 ml of concentrated shrimp nauplii in dechlorinated water to the treatment boxes. Control boxes received 10 ml of dechlorinated water.

The next morning, we collected the frog eggs laid overnight, placing approximately 20 sibling eggs into each experiment box. We randomly distributed 10 boxes per treatment per family across three shelves of a metal rack (*N* = 30 replicates per treatment). Eggs were kept at 23°C on a light–dark cycle of 14 h L : 10 h D. We removed dead shrimp from treatment boxes daily and replenished the boxes with new concentrated shrimp (treatment) or water (control) daily until the tadpoles hatched. All eggs hatched approximately 2 days later.

To assess whether shrimp exposure impacted the morphology of hatchlings, we left 15 hatchlings per box to develop for another project and randomly sampled the remaining hatchlings (*N* = 125 hatchlings per treatment). We euthanized the sampled hatchlings via immersion in a 0.1% aqueous tricaine methanesulfonate (MS-222) solution according to IACUC protocol ID 17-252.0-A and preserved them in 95% ethanol. We measured the preserved hatchlings using previously published methods [29]. Specifically, using hand-held digital callipers, we measured each hatchling's body size (snout–vent length; SVL) and orbitohyoideus (jaw) muscle width (OH; carnivores have larger OH). We have previously found that measurements taken with callipers are comparable to those taken with an ocular micrometer. We also measured the length of its gut (carnivores have shorter guts) by removing and uncoiling the intestines against a ruler. Finally, we determined its Gosner developmental stage [30] by visual inspection. Measurements were done blind with respect to treatment. We standardized OH width and gut length for body size by regressing ln(OH) and ln(gut length) on ln(SVL).

Because we were concerned that we did not have enough fairy shrimp in the first trial, we ran another trial in autumn 2022. This second trial differed from the first in the following three ways. First, two families of eggs were used from adults collected from the field (also near Portal, AZ) two months before the experiment began. Second, our shrimp treatments contained both fairy and brine shrimp (*Artemia* sp.). Brine shrimp are more easily raised at the high densities required to trigger carnivore development [31,32]; these brine shrimp were hatched from eggs in 25 ppt formulated seawater and fed a solution of baker's yeast. Third, we sampled up to four hatchlings from approximately 20 replicate boxes per treatment (control *N* = 66 hatchlings from 23 boxes, treatment *N* = 49 hatchlings from 17 boxes). Across the two trials, we had 47 replicate treatment boxes and 53 replicate control boxes across five families, representing 366 hatchlings measured.

To determine if early exposure to shrimp impacted the morphology of hatchlings, we used linear mixed-effects models in the R package ‘lme4’ [33]. Because there was no effect of trial, we pooled data across trials. Our response variables were SVL, Gosner stage, SVL-corrected jaw width and SVL-corrected gut length. Our fixed effect was treatment, and we specified the experimental box nested within the family as a random intercept. We used an *F*-test of fixed-effects terms with Kenward–Roger's method for denominator degrees of freedom for inference (‘lmerTest’ package; [34]). We performed all analyses in R v.4.1.2 [35].

## 3. Results

**Figure 2.**
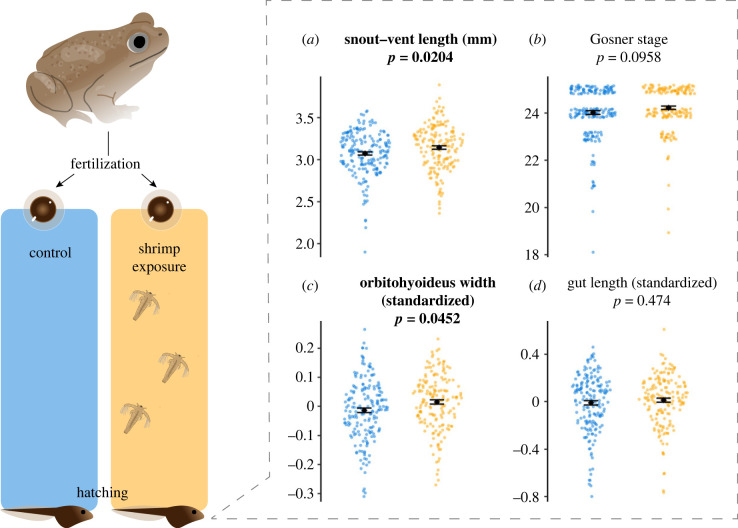
Response variables of *S. multiplicata* hatchlings that developed in shrimp nauplii's absence (blue dots) or presence (orange dots). Measurements of snout–vent length (SVL) (*a*), Gosner developmental stage (*b*), average width of the orbitohyoideus (jaw) muscle standardized by SVL (*c*) and gut length standardized by SVL (*d*). All panels show the individual data points (dots), where the width of the jitter represents the density distribution (mean ± s.e.m. are shown in black font). Traits in bold font represent significantly different groups (*p* < 0.05).

Hatchlings varied in morphology based on early (pre-feeding) exposure to shrimp (figure 2). Hatchlings with early exposure to shrimp nauplii were larger than control hatchlings (*F*_1,83_ = 5.627, *p* = 0.0204). However, shrimp-exposed and control hatchlings had a similar developmental stage (*F*_1,86_ = 2.837, *p* = 0.0958; most hatchlings were Gosner stage 24 or 25 at sampling). Hatchlings with early exposure to shrimp also had wider jaw muscles than control hatchlings (*F*_1,83_ = 4.131, *p* = 0.0452). However, shrimp-exposed and control hatchlings were similar in gut length (*F*_1,87_ = 0.516, *p* = 0.474). Finally, morphology varied by family (random effect variance for SVL: 0.0166, Gosner stage: 0.286, jaw: 0.00299, gut: 0.00284).

## 4. Discussion

Early developmental stages exposed to shrimp had larger body sizes and jaw muscles as hatchlings than those without such exposure (figure 2*a*,*c*). Although we could not ascertain if the cues from shrimp were detected by embryos, very early hatchlings or both, we can be confident that these differences were present at or within a few hours of hatching; i.e. before our experimental subjects had begun feeding. Moreover, such differences would have likely lasted until the hatchlings started feeding. Because young tadpoles that are larger outcompete smaller tadpoles for shrimp and thereby prevent smaller tadpoles from becoming carnivores [27], pre-feeding plasticity could prime those that express it to use the shrimp resource once they begin feeding. Additionally, tadpoles with larger jaw muscles create greater suction to intake prey [36], thereby having an advantage in eating shrimp [28]. Thus, pre-feeding developmental stages of *S. multiplicata* can assess and respond to shrimp in ways that likely jump-start resource-use plasticity, potentially providing hatchlings with a competitive advantage later in life [37].

Previous studies suggested meat consumption is necessary to induce a carnivore [18,24,25,32,38]. Yet, our data demonstrate that pre-feeding developmental stages respond to the mere presence of shrimp. Because none of the hatchlings in our experiment had begun feeding (and, indeed, we did not observe any changes in gut morphology; figure 2*d*), the morphological changes that we observed are indicative of anticipatory plasticity: shrimp exposure induced a phenotype with no current utility but one that is likely to be adaptive in the future. Although our use of the term ‘anticipatory plasticity’ differs from the usual definition in that, in our system, it is an anticipated *phenotype* rather than an anticipated environment [11], our results highlight that plastic resource-use phenotypes can be induced by cues other than direct ingestion. Given that resource-use plasticity is widespread [14–21] and thought to play an important role in many ecological and evolutionary processes [23], such anticipatory plasticity may be a crucial but understudied form of developmental plasticity.

Different sibships (i.e. different genotypes) varied in response to shrimp, suggesting underlying genetic variation in this plasticity. Such genetic variation in plasticity is common and could mediate adaptive evolution in response to different environmental conditions [39,40]. For example, genotypes highly responsive to shrimp cues early in development might be favoured when competition for shrimp is keen. Indeed, high shrimp density is a signal of pond ephemerality [18], and in such a pond, it is beneficial to develop into the more rapidly developing carnivore morph [18,26,41]. By contrast, in longer-lasting ponds with fewer shrimp, omnivores experience less severe competition for food and are, therefore, favoured over carnivores [42]. Substantial year-to-year and pond-to-pond variation in the intensity of resource competition exists in our study area [22,41,43,44] and could explain why such genetic variation might be maintained evolutionarily.

Our results suggest that shrimp provide a multifaceted cue to tadpoles across development. Pre-feeding stages detect and respond to a heretofore unknown chemical or tactile cue of shrimp presence by producing features as hatchlings that could enable individuals to take advantage of this nutritious resource once they begin feeding. At the same time, older tadpoles seem to require the consumption of large live animal prey to produce the distinctive carnivore morph [25,32]. This provides an intriguing example of an organism using separate subcomponents of a complex environmental cue differently across development.

Generally, when early life environmental cues are reliable, it should be beneficial for organisms to incorporate environmental information into their phenotypes early in development via anticipatory plasticity. Costs may be associated with remaining sensitive to environmental cues throughout life [45,46], so information received early in development can greatly impact later phenotypes [47]. Our study provides evidence for extremely early life assessment of environmental resources resulting in an adaptive plastic response, further emphasizing the critical role of developmental plasticity in shaping ecological and evolutionary dynamics.

## Data Availability

Data are available from the Dryad Digital Repository: https://doi.org/10.5061/dryad.hhmgqnkm9 [48]. Data were collected according to the methods described in the manuscript. Raw data are stored in csv files and described in a read-me file. Analysis was conducted using an R script.
